# Plant growth conditions alter phytolith carbon

**DOI:** 10.3389/fpls.2015.00753

**Published:** 2015-09-17

**Authors:** Kimberley L. Gallagher, Alba Alfonso-Garcia, Jessica Sanchez, Eric O. Potma, Guaciara M. Santos

**Affiliations:** ^1^Department of Earth Systems Sciences, University of California, IrvineIrvine, CA, USA; ^2^Department of Biomedical Engineering, University of California, IrvineIrvine, CA, USA; ^3^Department of Biology, California State University of FullertonFullerton, CA, USA; ^4^Department of Chemistry, University of California, IrvineIrvine, CA, USA

**Keywords:** phytolith, organic matter, Raman, silica, SRS, VCA

## Abstract

Many plants, including grasses and some important human food sources, accumulate, and precipitate silica in their cells to form opaline phytoliths. These phytoliths contain small amounts of organic matter (OM) that are trapped during the process of silicification. Previous work has suggested that plant silica is associated with compounds such as proteins, lipids, lignin, and carbohydrate complexes. It is not known whether these compounds are cellular components passively encapsulated as the cell silicifies, polymers actively involved in the precipitation process or random compounds assimilated by the plant and discarded into a “glass wastebasket.” Here, we used Raman spectroscopy to map the distribution of OM in phytoliths, and to analyze individual phytoliths isolated from *Sorghum bicolor* plants grown under different laboratory treatments. Using mapping, we showed that OM in phytoliths is distributed throughout the silica and is not related to dark spots visible in light microscopy, previously assumed to be the repository for phytolith OM. The Raman spectra exhibited common bands indicative of C-H stretching modes of general OM, and further more diagnostic bands consistent with carbohydrates, lignins, and other OM. These Raman spectra exhibited variability of spectral signatures and of relative intensities between sample treatments indicating that differing growth conditions altered the phytolith carbon. This may have strong implications for understanding the mechanism of phytolith formation, and for use of phytolith carbon isotope values in dating or paleoclimate reconstruction.

## Introduction

Many plants, including grasses and some important human food sources, accumulate, and precipitate silica in their cells and intercellular spaces to form opaline phytoliths. Silica deposition can be advantageous for the plant providing structural support, enhanced light penetration for photosynthesis, and resistance to herbivory and fungal attack (reviewed in Bauer et al., 2011). Phytoliths formed in specialized “silica cells” in the epidermis that fill with precipitated silica, can be diagnostic for their plant source based on their morphologies (e.g., Piperno, 2006). Phytoliths are well preserved in soils long after the plant dies and its biomass decomposes (Alexandre et al., 2011; Strömberg et al., 2013), and have been used extensively for paleoreconstructions based on their shapes (reviewed in Piperno, 2006; Santos et al., 2012). In addition, the isotopic composition of carbon occluded during precipitation within the silica matrix has been studied for information on climate change, community composition, and time of deposition (Wilding, 1967; Piperno and Becker, 1996; Piperno and Stothert, 2003; Carter, 2009; Webb and Longstaffe, 2010; McInerney et al., 2012).

Although the mechanism of phytolith formation has been studied for many years (early work is reviewed in Blackman, 1969, and see also Lawton, 1980; Hodson et al., 1985; Perry and Fraser, 1991; Sapei et al., 2007; He et al., 2013; Rudall et al., 2014), it is still poorly understood (Piperno, 2006; Song et al., 2012; Parr and Sullivan, 2014). According to Blackman (1969) and others, studies based on optical microscopy identified specialized silica cells, and showed that silica precipitation began at the periphery of senescing cells and was followed by infilling with silica that sometimes left vesicular cavities visible as dark spots.

An active process, such as templating onto lignified cell walls (Zhang et al., 2013) or onto proteins (Perry and Keeling-Tucker, 2000; Kauss et al., 2003; Currie and Perry, 2007; Elbaum et al., 2009), might result in enrichments of lignins or proteins relative to others in the silica matrix, and perhaps preservation of templating structures by the precipitated silica (Rudall et al., 2014). A passive mechanism would potentially encapsulate cell materials of all types (proteins, carbohydrates, lipids, etc.) if the silica precipitates while the cell is senescing and remnants of cellular structures are still present. Precipitation in a cell shaped void after senescence is complete might reflect the transpiration stream composition and include both plants metabolites, and dissolved soil components, as well as the chemical remnants of cellular structures (but not the structures themselves).

In a study on phytolith formation in *Equisetum arvense*, Perry and Fraser (1991) postulated that silica particles may nucleate in the transpiration stream and their nature may be controlled by physicochemical factors there, including, for example, silicon concentration and pH. Nucleation in the transpiration stream rather than in the silica cells might mean that occluded carbon reflects transpiration stream components. If so, the resultant isolated phytoliths would contain a transpiration stream OM signature. This source for phytolith composition is supported by elemental analysis of the inorganic constituents of silica phytoliths, which reveals that several different cations are present in the silica, most notably sodium, potassium, aluminum, calcium, and magnesium (Kamenik et al., 2013; Wu et al., 2014), and that the proportions more closely resemble terrigenous elements than cations of source plant material (Kamenik et al., 2013).

For organic matter, protein has been isolated from solubilized plant silica (Harrison, 1996) but DNA has not been identified (Elbaum et al., 2009). In a study using pyrolysis gas chromatography/mass spectrometry (pyGC/MS), supplemented by ^13^C nuclear magnetic resonance spectroscopy (^13^C-NMR); simple carbohydrates, and small amounts of alkanes and lipids were reported (Krull et al., 2003). The presence of fatty acid methyl esters, fatty alcohols, and straight chain hydrocarbons, indicative of lipids, has been shown in phytolith extracts using tetramethylammonium thermochemolysis (Smith and Anderson, 2001).

Raman Spectroscopy is an optical technique that is non-destructive and can be used to determine both the distribution and nature of chemical constituents in tissues. It has been used previously for analysis of lignin in plant cell walls (Agarwal and Ralph, 1997; Gierlinger et al., 2008) and for determination of silica distribution in plant tissues (Dietrich et al., 2003; Sapei et al., 2007; Blecher et al., 2012). The use of Raman spectroscopy for phytolith chemical analysis has been limited, perhaps because of the low amount of OM trapped within the silica (0.1% carbon for the samples used in this study Santos et al., 2014). Pironon et al. (2001) used Fourier transform infrared spectroscopy (FT-IR) combined with Raman spectroscopy to show changes in phytolith chemistry associated with heating phytoliths to 400°C for 10 min. This included changes in the silica, degradation of aliphatic carbon, and appearance of signals consistent with graphite. Watling et al. (2011) used a combination of Raman, Infrared and X-ray photoelectron spectroscopy to evaluate the efficiencies of different phytolith extraction methods (Watling et al., 2011). Their primary findings for the Raman spectroscopy were based on analysis of characteristic silica bands and not on OM content, although they did report Raman bands corresponding to cellulose at 2940 cm^−1^and bands at 2864 and 2880 cm^−1^ that were assigned to lignins. They obtained different results for different extraction methods.

Our study represents the first of its kind where Raman analysis of OM in phytoliths has been performed on plants grown with different soil treatments and extracted with the same protocol (Corbineau et al., 2013; Santos et al., 2014). Here, we map the distribution of OM in representative phytoliths and examine the phytolith OM composition using a new Raman imaging technology, called stimulated Raman scattering (SRS) microscopy, which has not previously been used for the study of phytoliths. Samples used in this study were obtained from the biomass of plants used in a previous isotope experiment designed to determine the influence of soil amendments on occluded organic matter in phytoliths (Harutyunyan et al., 2014; Santos et al., 2014). In that study, radiocarbon values were determined, but the structural composition of the occluded carbon was not. The authors speculated there was a soil carbon source for the phytolith OM based on radiocarbon results, and demonstrated a correlation between soil amendments and phytolith radiocarbon dates; however, a soil carbon contribution to phytolith OM remains controversial. Previous authors have maintained that phytolith OM is derived only from host plant tissue, and therefore it can be used for paleoreconstructions and paleoclimate studies (reviewed in Santos et al., 2012). Indeed, the source of phytolith OM may be host plant tissue, some component of the soil amendment, microbially reworked low molecular weight OM, dissolved inorganic carbon, or some combination of the above. A better understanding of the nature of the occluded organic matter in phytoliths, and whether it changes depending on growth conditions, may support a hypothesis of a soil organic matter contribution.

## Materials and methods

### Sample preparation

Samples were obtained from a prior study (Santos et al., 2014) designed to investigate the uptake of soil OM and its potential effect on phytolith radiocarbon dates. In that study, *Sorghum bicolor* was grown in planters outdoors with commercial amendments according to the scheme in Table 1 (More details of the commercial amendments and their use is available in Tables S1, S2). The treatments were designed to provide soil amendments with an array of isotopically pre-characterized carbon. In addition, inorganic commercial products containing trace carbon were used for the other samples (i.e., the substrate used in Treatment B, and the fertilizer used in Treatments B, C, D, and E; (Table S1). Sample F was the designated experimental control since it was initially free of organic carbon additives. Phytoliths used in our study were extracted and purified from the plant leaves and stems according to the wet oxidation procedure described in Corbineau et al. (2013). Additionally, phytoliths from a *Sorghum* field study (designated as Sample S; Ottman et al., 2001) and from a volcanic soil (Sample M; Meunier et al., 2010) were included for comparison. Sample M (MSG70) was obtained from natural soils and was not a characteristic *Sorghum bicolor* phytolith, but a mixture of phytoliths from other plants (Meunier et al., 2010). The carbon and nitrogen composition of the trapezoidal shape in this sample was evaluated previously using nanoSIMS (Secondary ion mass spectroscopy; Alexandre et al., 2015).

**Table 1:** **Experimental treatments for six different planters (A–F)**.

**Experimental treatment**	**A**	**B**	**C**	**D**	**E**	**F**
Substrates	Potting soil	Nonsterile sand	Sterile sand	Sterile sand	Sterile sand	Sterile sand
Organic Amendments	Compost	No organic additive	No organic additive	Kelp meal	Humic acids	No organic additive
Inorganic Fertilizer	NH_4_NO_3_, (NH_4_)_3_PO_4_, Ca_3_(PO_4_)_2_, K_2_SO_4_	Ca(NO_3_)_2_, KNO_3_, H_3_PO_4_, HNO_3_, K_2_SO_4_	Ca(NO_3_)_2_, KNO_3_, H_3_PO_4_, HNO_3_, K_2_SO_4_	MgSO_4_ borax, CoSO_4_, FeSO_4_, MnSO_4_, Na_2_MoO_4_, ZnSO_4_ NaH_2_PO_4_, MgSO_4_, Ca(NO_3_)_2_ KNO_3_	NaH_2_PO_4_, MgSO_4_, Ca(NO_3_)_2_ KNO_3_	NaH_2_PO_4_, MgSO_4_, Ca(NO_3_)_2_ KNO_3_
Silica amendment	No added silica	No added silica	Na_2_SiO_3_, K_2_SiO_3_	Na_2_SiO_3_, K_2_SiO_3_	Na_2_SiO_3_, K_2_SiO_3_	Na_2_SiO_3_, K_2_SiO_3_

Note especially the differences in OM additives. Ingredients reported here were obtained from manufacturers' descriptions of commercial products. Planters A-E utilized commercial products that contained traces of carbon. Planter F was an inorganic control that did not contain trace carbon in the soil amendments (adapted from Harutyunyan et al., 2014). Samples S and M (not shown) are comparison samples from a field study and a common standard phytolith collection, respectively and are not part of the study outlined above. See text for more information.

### Scanning electron microscopy (SEM)

SEM was performed on extracted phytoliths from Sample E to demonstrate the variety of silica structures obtained in the isolation protocol. A sample was mounted on carbon tape on an aluminum stub and sputter coated (Polaron SC7620, Quorum Technologies, Newhaven, East Sussex, England) with gold/palladium to a thickness of ~3 nm. Microscopy was performed on a Phillips XL30 Field Emission Scanning Electron Microscope (FEI Company, Hillsboro, OR, USA) at an accelerating voltage of 2 kV. Planter E was chosen to demonstrate the phytolith morphology because it gave the highest yield of sample material and also exhibited a large variety of silica forms.

### Raman spectroscopy

**Figure 1:**
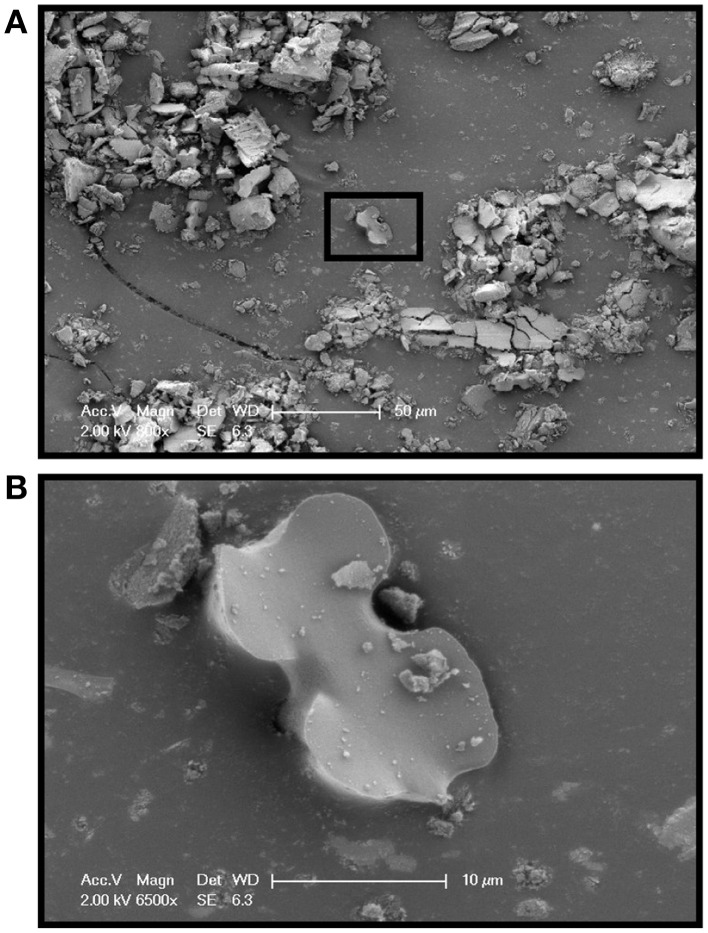
**Scanning electron micrographs showed that phytoliths obtained from the extraction exhibited a variety of shapes consistent with silica deposition both within and between cells (A, 200 μm scale bar)**. For consistency, only the bilobate morphology, consistent with silica precipitated within cells (**B**, 10 μm scale bar) was used for this study. Phytoliths from sample treatment E are shown here.

The phytolith samples exhibited a variety of morphologies based upon the cells or tissues in which the silica precipitated (Figure 1). Atypical silica bodies (not originating from specific silica cells) are well-known (Blackman, 1969), and were most notable in samples with higher silica inputs (Treatments C, D, E, and F) where intercalated silica sheets, casts of stomata, and broken pieces were observed. For consistency in our study, only bilobate phytoliths (30 phytoliths per treatment x 6 treatments; Figure 1B), typical of *Sorghum bicolor* silica cells, were randomly chosen in each sample for Raman analysis. For the MSG70 soil phytoliths only the trapezoidal shape, typical of a grass short cell was evaluated (*n* = 30 phytoliths).

#### Mapping with stimulated raman scattering (SRS) microscopy

To study the spatial distribution of the OM entrapped within the phytoliths we used SRS microscopy. SRS allows fast and label-free acquisition of images with contrast derived from the Raman-active molecular vibrations of the sample (Freudiger et al., 2008; Nandakumar et al., 2009; Chung et al., 2013). Images of the phytoliths were acquired with a custom SRS microscope interfaced with two ps laser beams: the so-called pump and Stokes beams. A 76 MHz mode-locked Nd:Vanadate laser (Picotrain, High-Q, Hohenems, Austria) delivered a fundamental beam at ~9400 cm^−1^ (ω_S_, the Stokes beam) with 7 ps pulses, and a second harmonic generated beam that pumped an optical parametrical oscillator (OPO; Levante Emerald OPO, Berlin, Germany). The OPO emitted the pump beam, ω_p_, which can be tuned to probe different molecular vibrational bands, ω_vib_ = |ω_p_ – ω_S_|. The Stokes and pump beams were overlapped in space and time and directed to an inverted microscope (IX71, Olympus, Center Valley, PA). The combined laser beams passed through a laser scanner (Fluoview 300, Olympus) and were focused by a 20 ×, 0.75 NA objective lens (UPlanSApo, Olympus) onto the sample. The Stokes beam was modulated at 10 MHz with an acoustic-optical modulator (Crystal technology, LLC). Average illumination power at the sample was up to 15 mW per beam. The transmitted pump beam was detected with a photodiode (FDS1010, Thorlabs) and demodulated with a custom lock-in amplifier. Hyperspectral images of the phytoliths were acquired to reconstruct the CH stretching region of the Raman spectra (2800–3100 cm^−1^) as previously described (Lim et al., 2011). Image acquisition times were typically 1 s/frame (512 × 512 pixels), while acquisition of a hyperspectral data cube was on the order of 30 min (up to 40 spectral points). The obtained Raman maps were analyzed with vector component analysis (VCA; Hedegaard et al., 2011).

#### Spectral comparison

Dry mounted samples (in air) were analyzed using a commercial Raman microscope (InVia Confocal; Renishaw, Wotton-under-Edge, Gloucestershire, UK). Spectra were taken using a 532 nm laser with a 2400 l/mm grating, and a 50 × 0.75 NA objective lens. The laser power at the sample plane was 30 mW. Raman spectra were collected from a diffraction limited focal spot within the phytolith at an exposure time of 5 s and 10 accumulations. Two spectral windows were analyzed, one in the CH stretching region from 2700 to 3150 cm^−1^, and another in the fingerprint region from 1250 to 1800 cm^−1^. Other typical diagnostic regions were unavailable because of the presence of overwhelming silica bands. The spectra were first corrected by manually subtracting their autofluorescent background, and then smoothed with a Savitsky-Golay filter implemented in Matlab. For most of the analysis, the spectra of each spectral window were normalized to the highest intensity peak independently. For the variability analysis between samples, the positions and relative intensity of each Raman band were taken to generate plots with the average spectrum and standard deviations (±1) of each group of 30 samples per planter. Comparison of peak intensities was performed using non-normalized spectra (**Figures 4A,B**). In addition, a direct comparison of the relative intensity of the Raman bands 1603, 2907, and 3074 cm^−1^ was also performed between the different planter groups. A Mann-Whitney *U*-test determined the groups with statistically significant means (^*^p < 0.05; **Figure 4C**).

## Results

### Mapping

**Figure 2:**
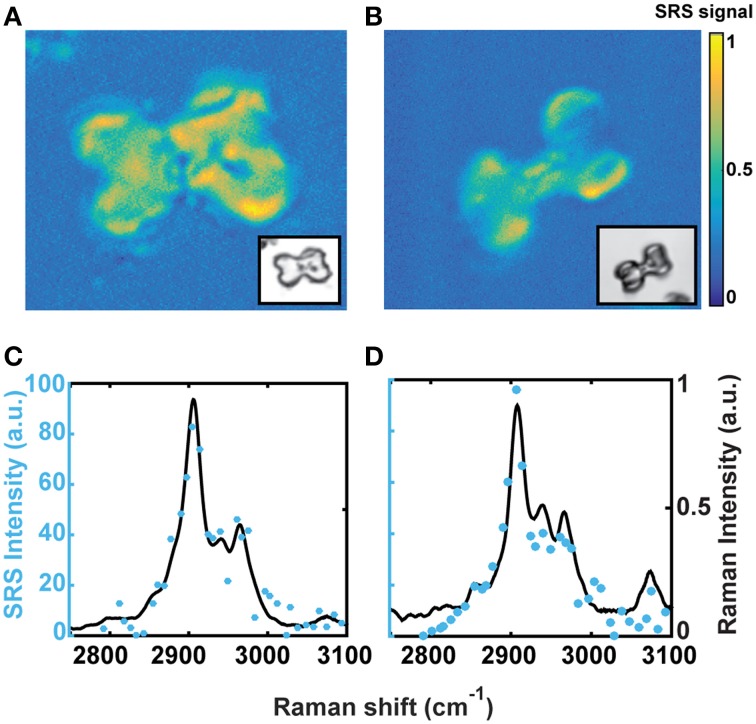
**Hyperspectral imaging of individual phytoliths from samples A and E shows OM is distributed unevenly throughout the silica matrix**. Dark spots visible in optical microscopy do not contain OM and there are no obvious patterns in the OM distribution. **(A, B)** Show one end-member of the VCA for phytolith A and E hyperspectral imaging. Insert is the corresponding optical image. **(C, D)** are the Raman spectra in the CH region (solid line is average of all spectra, blue dots are specific SRS intensity measures).

SRS microscopy was used to acquire chemical maps that revealed the spatial distribution of the OM within a phytolith. Hyperspectral imaging of a phytolith of planters A and E was analyzed by vertex component analysis (VCA). The resulting intensity maps (Figures 2A,B) show that the OM were inhomogeneously distributed throughout the phytoliths. Although the distribution was somewhat irregular, the OM was found to occupy the entire volume of the phytolith with no particular discrete site for OM concentration. The yellow areas correspond to regions of the phytolith with higher concentration of OM, and have Raman spectra (blue dots) similar to the mean Raman spectra (black line) for each group (Figures 2C,D). There was no OM detected in the dark spots visible by light microscopy, supporting previous nanoSIMS work by Alexandre et al. (2015).

### Spectral comparison

**Figure 3:**
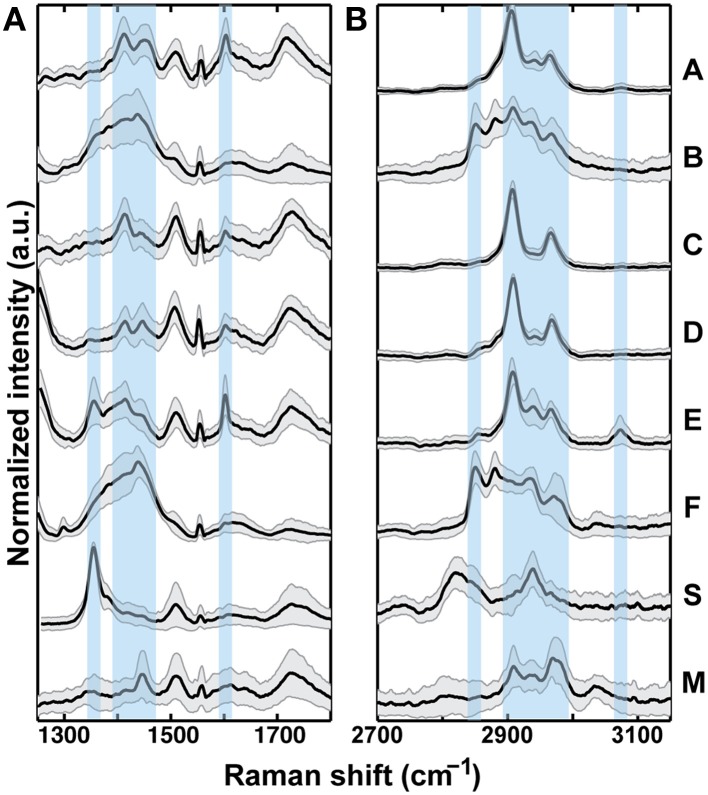
**Raman spectroscopy results for bilobate phytoliths from treatments A–F, and S, and trapezoidal phytoliths from M**. Variability in peak locations and relative intensities, designated by vertical bars, indicate locations of Raman band disparities. Spectrum averages (heavy line) and variability (gray area, ±1 standard deviation) within each sample (*n* = 30) in the fingerprint region **(A)** and the CH region **(B)** are shown. Samples B, F, S, and M exhibited the largest standard deviation from their average spectra.

Raman bands and intensities varied between sample treatments (Figure 3). In the fingerprint region (1250–1800 cm^−1^), overlapping modes are present and can be assigned to various CH bonds especially in the 1350–1450 cm^−1^ range (Table 2). A strong peak at 1356 cm^−1^ in Sample S is also present in Sample E (Figure 3, highlighted) and could represent tertiary CH groups or NO bonds. The peak at 1500 cm^−1^ (not highlighted) is present in the glass sample (i.e., originating from the microscope slide on which the phytoliths were mounted) and is not attributed to OM. A band corresponding to aromatic ring stretching at 1603 cm^−1^ (highlighted) is seen clearly in Samples A and E, and is also present in Samples C and D to a lesser extent. In the CH stretching vibrational region (2700–3150 cm^−1^), all samples exhibited peaks indicative of some OM (highlighted region 2900–3000 cm^−1^), although the relative intensities and the overall shape of the spectra varied indicating different OM contributions. Samples B and F appeared to be most distinct from the others and both exhibited a strong peak at 2850 cm^−1^ (highlighted) that is not present in the other samples. A Raman band at 3073 cm^−1^ that is present in both samples A and E can be assigned to the carbon-hydrogen stretching mode of the CH = CH- group, and signifies the presence of unsaturated hydrocarbons.

**Table 2:** **Summary of Raman band locations and assignments**.

**A**	**B**	**C**	**D**	**E**	**F**	**S**	**M**	**Range(where present)**	Peak assignments^*^
–	–	–	–	–	1299	–	–	1299	(CH_2_)_*n*_ deformations
–	–	–	–	1356	–	1355	–	1356	Tertiary (CH) deformation
1411	1415	1413	1413	1413	1415	–	–	1411–1415	(CH_2_) or (CH_3_)
–	1438	–	–	–	1439	–	–	1438–1439	(CH_2_)
1450	–	1443	1447	1447	1449	–	1445	1443–1450	(CH_2_), (CH)
1509	–	1509	1507	1511	–	1510	1510	1507–1511	Asymmetrical aryl ring stretch
1603	–	1603	1603	1602	1605	–	–	1602–1605	Aryl stretching vibration
1716	1724	1729	1724	1727	1719	1727	1728	1716–1729	C = O stretch of aldehydes, ketones, esters
–	–	–	–	–	–	2820	–	2820	CH bending
–	2851	2845	2856	2856	2850	–	–	2845–2856	CH antisymmetric and symmetric stretch of aliphatic compounds
–	2882	–	–	–	2881	–	–	2881–2882	CH antisymmetric and symmetric stretch of aliphatic compounds
2905	2907	2907	2908	2907	–	–	2909	2905–2909	CH antisymmetric and symmetric stretch of aliphatic compounds
–	2932	–	–	–	2932	–	2934	2932–2934	CH antisymmetric and symmetric stretch of aliphatic compounds
2939	–	–	2941	2938	2938	2939	–	2938–2941	Antisymmetric C–H stretch in –OCH_3_ symmetric CH stretch
2963	2966	2966	2968	2966	2967	–	2969	2963–2969	CH antisymmetric and symmetric stretch of aliphatic compounds
3074	–	–	–	3073	–	–	–	3073–3074	(=C–H)

The phytoliths from these samples showed many common OM bands. Planters B and F exhibited a different pattern from the others in the growth experiment. Samples S and M were included for reference.

* Lambert et al., 1998.

Variability within samples was determined by the ±1 standard deviation spread with respect to the average spectra collected from *n* = 30 phytoliths. Generally, Raman spectra of different phytoliths within each sample varied the most in the fingerprint region and less in the CH region (Figure 3). In addition, the CH stretching regions of samples B, F, S, and M were generally more variable than in A, C, and D. In the samples exhibiting higher variability it was more common for a randomly chosen sub-sample to have little to no signal (Complete 30 spectra are shown in Figure S3).

**Figure 4:**
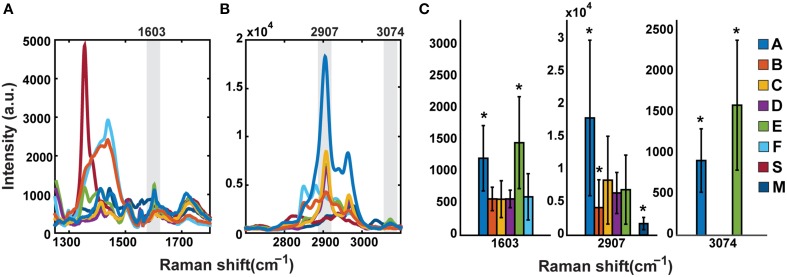
**Comparison of mean non-normalized signal intensities shows variability by sample treatment**. Samples A, C, D, and E show common features both in the fingerprint region **(A)** and the CH stretching region **(B)**. Sample M is less similar but retains some characteristics of the above. Samples B and F show a broad higher intensity set of peaks in the fingerprint region and lower intensities in the CH stretching region from the other samples. **(C)** is the statistical comparison of peaks 1603, 2907, and 3074 cm^−1^. Sample A has the highest relative intensity at 2907 cm^−1^. Asterisks denote statistical differences (^*^p < 0.05).

A comparison of non-normalized average peak intensities in the fingerprint region for samples A–F is given in Figure 4A. The aromatic band intensity at 1603 cm^−1^ is strongest for samples A and E. Samples B and F show a strong and broad contribution in the 1300–1500 cm^−1^ region, which is not attributed to group vibrations of OM and likely originates from inorganic materials. In the CH region (Figure 4B), the 2907 cm^−1^ peak intensity for sample A is 2–3 times higher than C, E, and D. Although B and F displayed the highest intensity peaks in the fingerprint region, their peaks in the CH stretching region, though more numerous, were less intense. Only samples E and A show a peak at 3074 cm^−1^ (unsaturated CH). A graph showing the relative intensities of the assigned bands at 1603, 2907, and 3074 cm^−1^ is also shown for clarity (Figure 4C). Mann-Whitney *U*-test (^*^p < 0.05) reveals groups A and E have a significantly larger mean than the rest of the groups at the 1603 cm^−1^ Raman band, but not between each other. For the band at 2907 cm^−1^, the Sample A mean is significantly larger than all the rest, and Samples B and M means are significantly smaller. Sample S shows no peak at this position. Finally, only Samples A and E have a peak at 3074 cm^−1^, with significantly different means.

## Discussion

In this study, SRS hyperspectral imaging in combination with Raman spectroscopy was performed to determine OM distribution, and signal intensities of average spectra were compared. Phytoliths from plants grown under different conditions contained varied carbon contents according to their Raman bands and intensities (Figure 3). Comparisons were made both between samples and within samples.

### Hyperspectral imaging

Cell wall templating as discussed above should result in evidence of cellulose and perhaps lignin at the periphery of the phytoliths (Zhang et al., 2013). Mapping did not show any evidence of this, although it cannot be ruled out as the sample preparation could have degraded the outer layers of the phytoliths. Remnants of cellular organelles were also not evident in the mapping results. Cellular organelles are rich in protein and would have resulted in distinct Raman spectral signatures. We find no evidence of such spectral signatures, indicating that cellular organelles cannot be the primary source of the observed OM in the silica matrix. In addition, there were no discrete organic structures within the silica matrix or within the dark spots visible in optical microsopy (Figure 2). The dark spots were devoid of OM, corroborating recent nanoSIMS findings by Alexandre et al. (2015). This indicates that silicification likely occurs in cells that have completely senesced. Based on the VCA analysis of the hyperspectral SRS imaging in the CH region (Figure 2), the OM is distributed throughout the silica matrix. This is consistent with entrapment of dissolved OM, originating either from the transpiration stream or from the completely degraded cellular materials, but not entrapment of cellular structures. This is consistent with previous observations and hypotheses of phytolith formation (Lawton, 1980; Hodson et al., 1985; Perry and Fraser, 1991; Sapei et al., 2007; He et al., 2013; Rudall et al., 2014),

### Comparison between samples

The biggest differences within the growth experiment samples were noted comparing samples B and F to other samples. Both of these were grown in sand with no particular organic media added to the soil, although it should be noted that the planters were not sealed throughout the course of the experiment and it is possible that organic carbon could have been introduced outside the experimental parameters (i.e., birds, bugs, pollen, etc.). Additionally, some of the commercial substrates and amendments contained trace organic carbon as supplied (Table S1), particularly the substrate used in Planter B (Jersey Greensand, Fertrell, Bainbridge, PA). The carbon may be organic or inorganic. According to Table 1, sample C should have given results similar to Sample F, but it most closely resembles samples A, D, and E (Figure 3). Sample B, which has trace organic carbon in the substrate, yields a spectrum resembling the inorganic control F. The broad combination of peaks obtained in samples B and F in the fingerprint region was distinctly different from the more individualized bands in samples A, C, D, and E. The signal in the 2800–3150 cm^−1^ region for samples B and F also differed markedly from other samples in having more peaks, and lacking the strong band at 2907 cm^−1^ that was present in the other samples.

For comparison with our experimental samples we also included samples from two other sources. The *Sorghum* biomass for Sample S came from the ambient plot used as a control for a study of atmospheric CO_2_ enrichment (Ottman et al., 2001). In the fingerprint region Sample S showed a band at 1350 cm^−1^ that was much stronger than other bands in its spectrum. Although this peak coincides with the vibrational frequency of a tertiary CH group (Table 2), the lack of other diagnostic organic peaks in this sample does not strongly support this assignment for Sample S. Another possibility is an NO bond originating from inorganic soil nitrogen, possibly as nitrates. In a previous study a Raman band at 1350 cm^−1^ was assigned as a graphite peak caused by prolonged high heat conditions (Pironon et al., 2001), but our samples were all prepared under identical conditions (Santos et al., 2014), and so it seems unlikely that some would contain graphite while some would not. Additionally, the presence of graphite should be corroborated by another strong Raman band at 1580 cm^−1^ that is not present. Sample M, obtained from volcanoclastic soils (Meunier et al., 2010), had an average Raman spectrum that was more similar to our planter samples than Sample S, although the signal intensity was low and samples were more variable (Figure 3). OM was detected in all samples and Raman spectroscopy revealed differences in the composition. Few specific compound assignments can be made because of the complexity of the overlapping peaks; however, in the CH stretching region, there was strong evidence of carbohydrates (Pizzini et al., 1988), particularly in Samples A, C, D, and E. Other samples were not as definitive.

### Comparison within samples

Variability within samples was observed primarily as variations in peak intensities, though slightly different spectra within samples were sometimes obtained (Figures S1–S8). In some samples it was common to find phytoliths with little or no signal. Differences may have been caused by heterogeneity at the molecular scale, i.e., our probing spot simply missed the OM in some sub-samples. This effect may have been exacerbated if the quantity of OM in some was less than in others. We found more variability in samples B, F, S, and M.

### Signal intensity

For comparison of quantities of OM in the samples, we examined the mean non-normalized spectra (Figure 4). Here the direct comparison of the spectra confirms the differences between the treatments especially the very broad and intense peak(s) of B and F in the fingerprint, and the relatively higher intensity of the peaks in Samples A and E in the CH stretching region, as exemplified by the larger 2907 cm^−1^ band. The strong signal in Sample A is very interesting from a mechanistic point of view as well as for comparison between samples. Sample A was extracted from biomass grown in potting soil with no additional silica amendment [aside from the small amount included in the commercial potting soil (Miracle Gro® Potting Mix, Scotts Miracle Gro, Marysville, OH)]. Therefore, the amount of silica available to the plant was much less than in samples grown in silica sand (and sometimes supplemented with extra silica, Table 1). The phytolith yield for A was correspondingly low. Harutyunyan et al. (2014) reported that as a percent of dry biomass, the phytolith yield of sample A was 0.12% compared to 0.78, 0.83, 0.83, 1.77, and 1.35% for the other experimental treatments, samples B–F respectively. Yields of samples S and M were not given.

**Figure 5:**
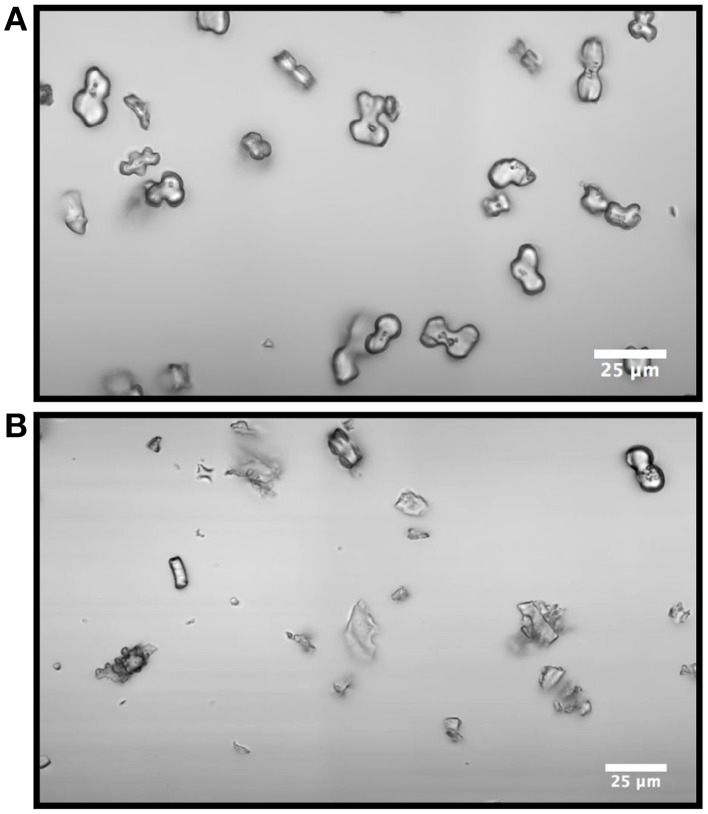
**Optical image of bilobate phytoliths and other silica debris from Samples A (A) and E (B)**. Sample E contains more silica remnants of other tissues. Scale bar = 25 μm.

In addition, for Sample A the proportion of bilobate phytoliths to other silica shapes was higher than other samples as exemplified by a qualitative comparison of images from Samples A and E (Figure 5). The relatively high signal intensity in A compared to other samples could represent a concentration effect because the amount of silica in the transpiration stream would be lower in proportion to the organics, or it could relate to speed of precipitation and entrapment of OM into the amorphous silica (Blackman, 1969; Hodson et al., 1985). The bands cannot be assigned to a particular type of organic compound, though the pattern in the CH stretching region is consistent with that of a carbohydrate (Wiley and Atalla, 1987; Pizzini et al., 1988). Enhanced carbohydrate signals from polysaccharides such as cellulose can be related to the mechanism of precipitation. It has been proposed previously that silicification is preceded by lignification of plant cell walls (Zhang et al., 2013; Rudall et al., 2014) which would perhaps result in enriched carbohydrate and lignin signals. We examined the putative carbohydrate peak at 2907 cm^−1^ and the characteristic lignin peaks at 1603 cm^−1^ and 3074 cm^−1^ (Figure 3C), and found different intensities between the samples. The Raman band at 1603 cm^−1^ consistent with lignin aromatics is corroborated by the unsaturated CH stretching band at 3074 cm^−1^ (Agarwal and Ralph, 1997).

### Carbon source and implications

The original motivation for this study was derived from attempts to explain anomalous radiocarbon results in phytolith deposits (Santos et al., 2014). It was proposed that a soil contribution to phytolith OM could be the cause of these anomalies (Santos et al., 2012). Earlier assumptions of phytolith OM were at least partly based on entrapment of cellular organelles and other plant tissues, reviewed in Santos et al. (2012). We did not see evidence of any discrete OM structures in our hyperspectral imaging. Furthermore, in our samples, the dark spot commonly observed in phytoliths under light microscopy was devoid of OM. While these findings do not confirm a soil OM contribution, they also do not support a hypothesis of trapped cellular organelles. In addition, according to our results, phytoliths grown with different soil media contain different OM residues within their silica matrices. This supports a soil contribution to phytolith carbon, although it is possible that physiological conditions of the plant (and by extension, plant metabolites in the transpiration stream) may also be altered by changing growth conditions. For radiocarbon dating, the effect of a soil contribution would be problematic given that natural soil OM is heterogeneous with respect to composition and age (Schmidt et al., 2011). Further experiments using fully characterized soil OM, perhaps combined with analyses of the transpiration stream composition and subsequent observation of phytolith OM, would be needed to more fully answer whether soil OM is contributing to phytolith OM.

## Conclusions

Here we have shown that OM is distributed throughout the silica matrix of phytoliths and is not present as discrete areas, supporting recent nanoSIMS results (Alexandre et al., 2015). We have also shown, for the first time, that Raman spectra of phytolith OM changes depending on plant growth conditions. Further studies are needed to fully determine the cause of these OM differences and whether they are from soil OM contributions or from metabolic changes in the plant.

### Conflict of interest statement

The authors declare that the research was conducted in the absence of any commercial or financial relationships that could be construed as a potential conflict of interest.

## Supplementary material

Figures S1–S8 **Raman spectra for 30 measurements of treatments A–F, S, and M showing variability between the individual phytoliths**. Note the scale difference between the growth experiment samples (A–F) and the reference samples S and M reflecting the lower peak intensities obtained in S and M.

Table S1 **Details of substrates and soil amendments including experimentally determined carbon content (adapted from Harutyunyan et al., 2014)**.

Table S2 **Planter treatments by commercial amendments (adapted from Harutyunyan et al., 2014)**.
