# Supplementary figures and images for: Plant growth conditions alter phytolith carbon
Source: Front Plant Sci. 2015 Sep 17;6:753. doi: 10.3389/fpls.2015.00753 (PMC4585121; doi:10.3389/fpls.2015.00753)

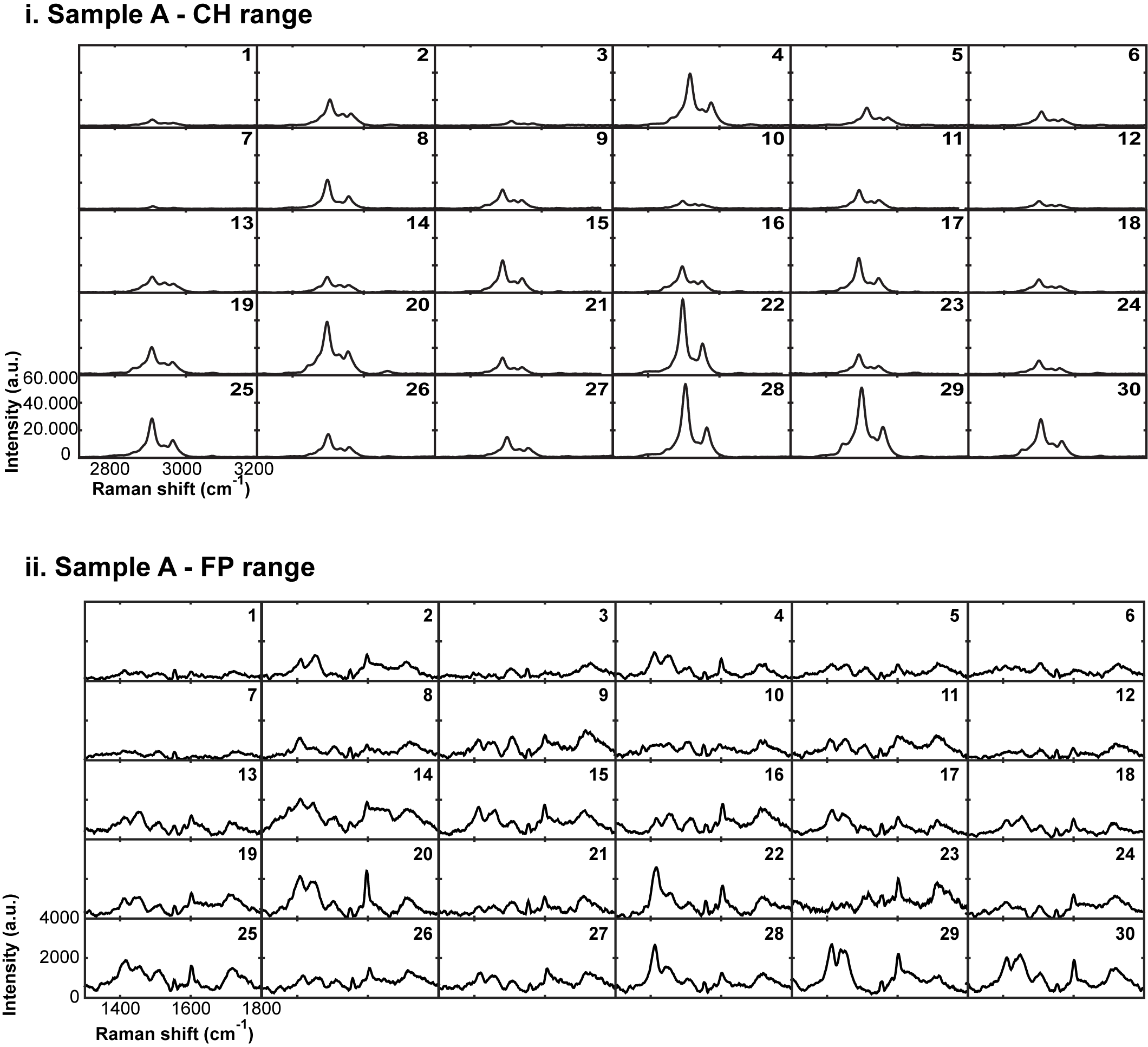

Supplement: Supplementary file 1 [file Image1.TIF]

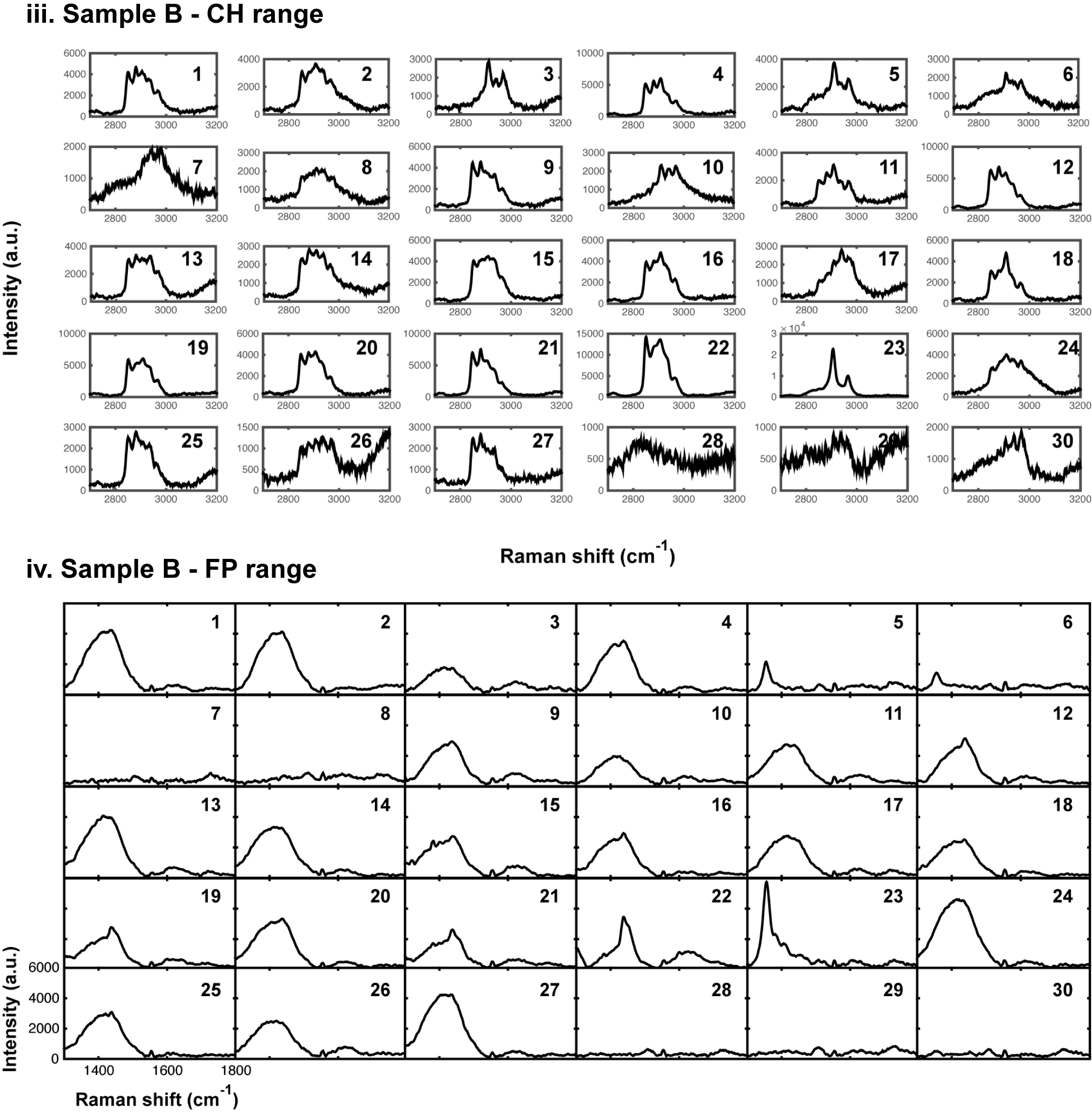

Supplement: Supplementary file 2 [file Image2.TIF]

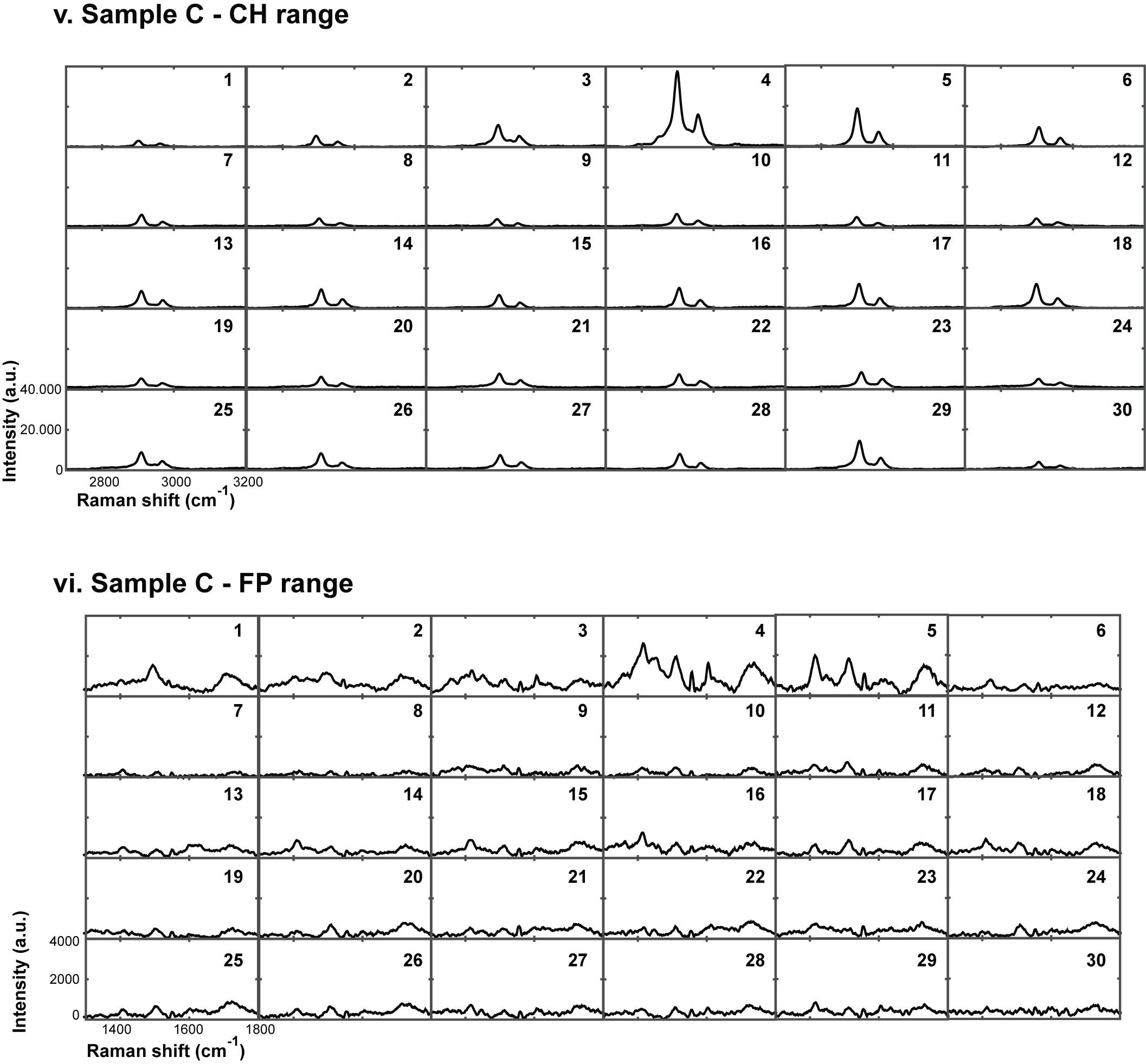

Supplement: Supplementary file 3 [file Image3.TIF]

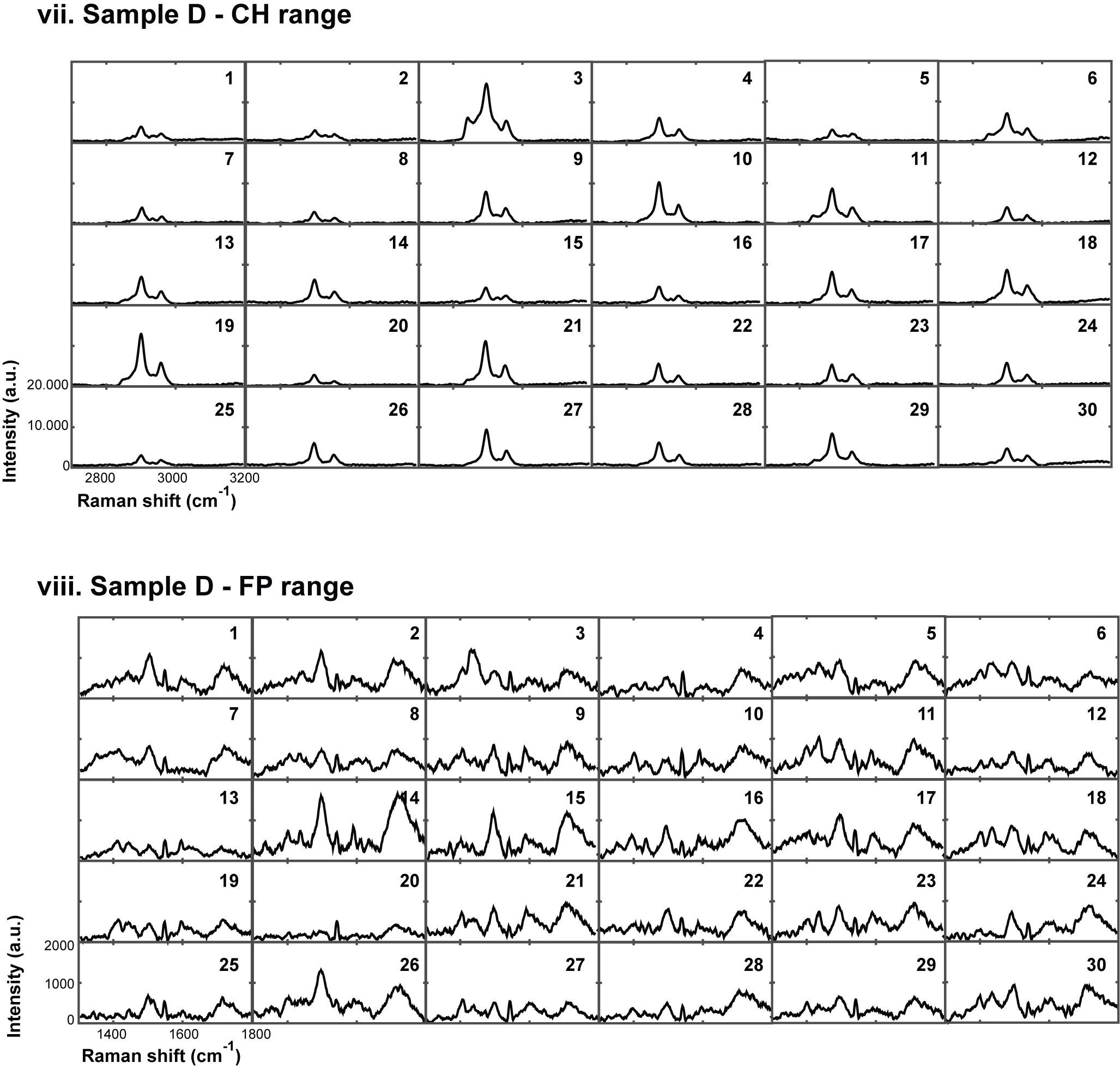

Supplement: Supplementary file 4 [file Image4.TIF]

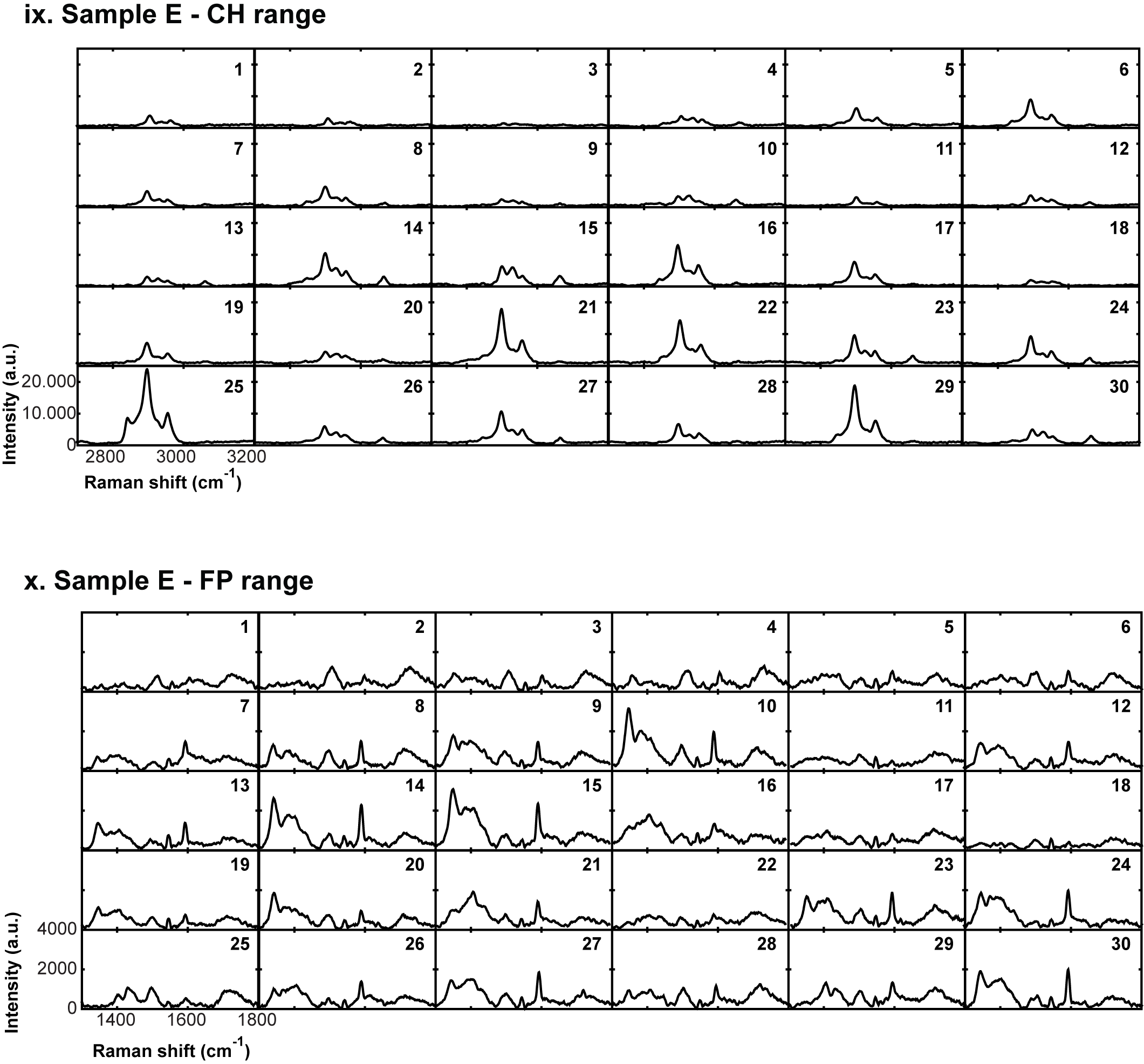

Supplement: Supplementary file 5 [file Image5.TIF]

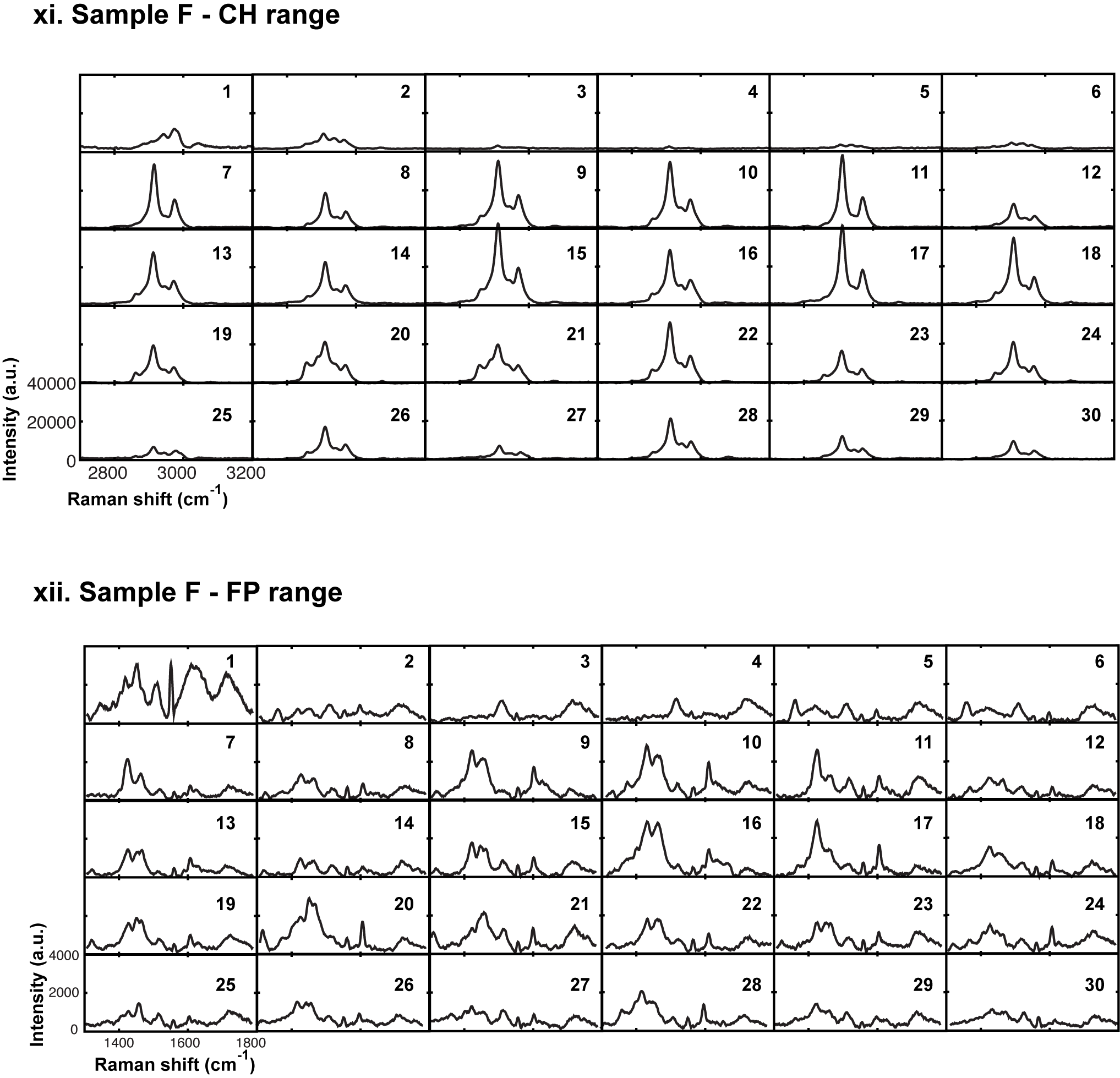

Supplement: Supplementary file 6 [file Image6.TIF]

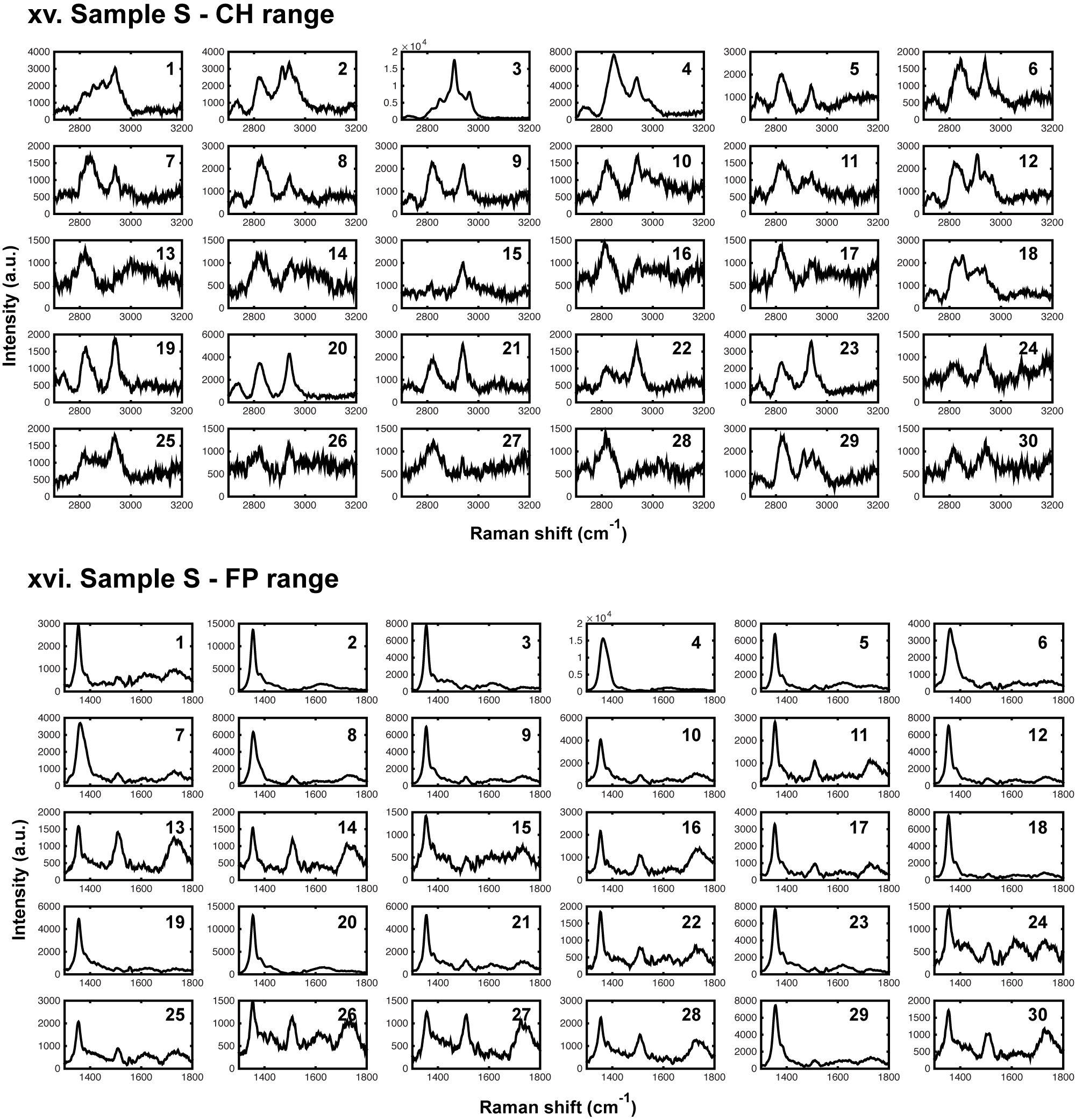

Supplement: Supplementary file 7 [file Image7.TIF]

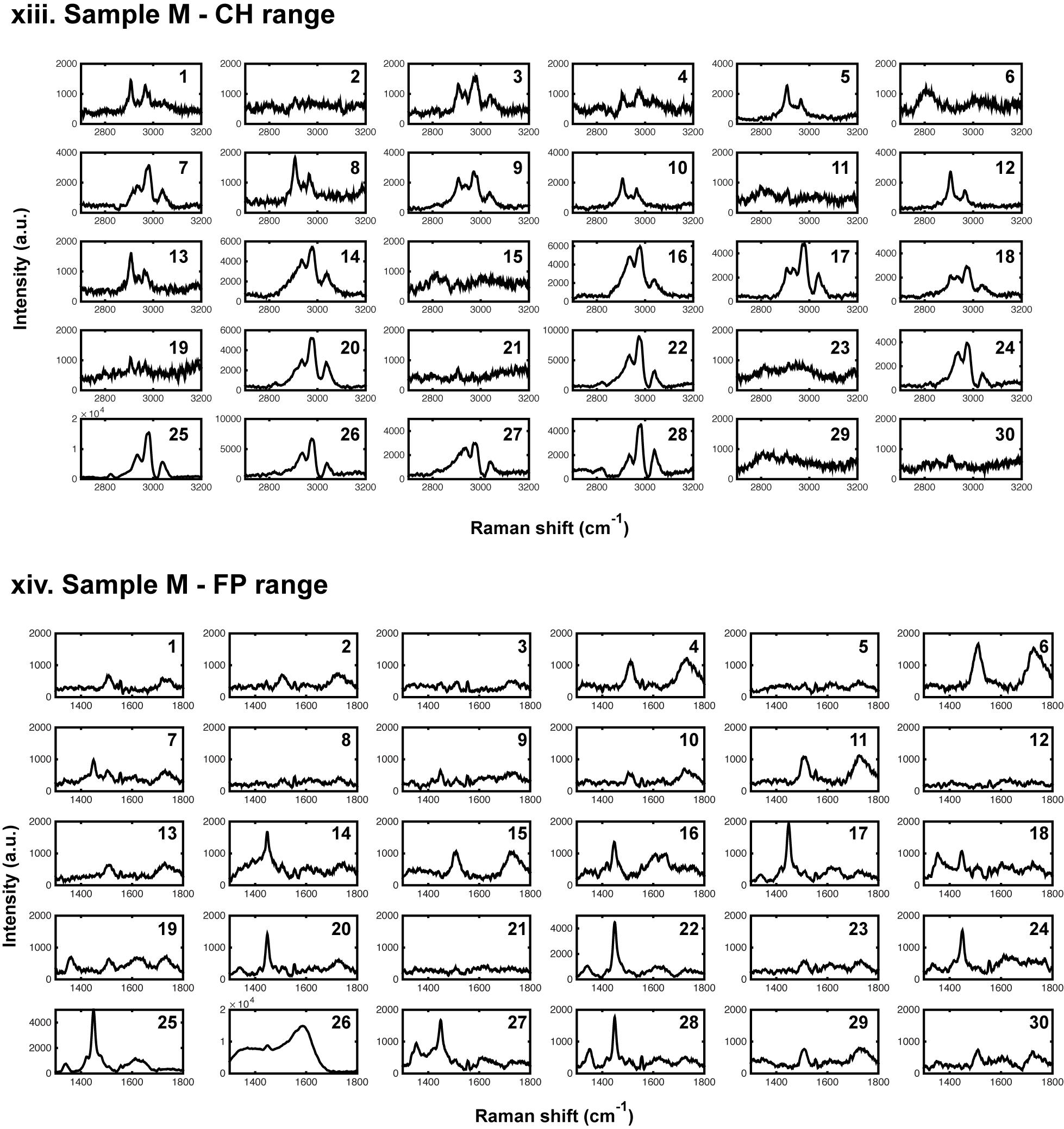

Supplement: Figures S1–S8 — Raman spectra for 30 measurements of treatments A–F, S, and M showing variability between the individual phytoliths. Note the scale difference between the growth experiment samples (A–F) and the reference samples S and M reflecting the lower peak intensities obtained in S and M. [file Image8.TIF]
