# Supplementary material for: Plant growth conditions alter phytolith carbon
Source: Front Plant Sci. 2015 Sep 17;6:753. doi: 10.3389/fpls.2015.00753 (PMC4585121; doi:10.3389/fpls.2015.00753)
Supplement: Table S1 — Details of substrates and soil amendments including experimentally determined carbon content (adapted from Harutyunyan et al., 2014). [file Table1.DOC]

Table S1: Details of substrates and soil amendments including experimentally determined carbon content (Adapted from Harutyunyan et al. (2014).

| ID | Commercial Name | Manufacturer/U.S. Headquarters | Manufacturer’s Short description | %C as supplied (mass) |
| --- | --- | --- | --- | --- |
| MG | Miracle Gro® Potting Mix | Scotts Miracle Gro, Marysville, OH | *Sphagnum Moss, Perlite, Compost, NH_4_NO_3_, (NH_4_)_3_PO_4_, Ca_3_(PO_4_)_2_, K_2_SO_4_* | 49.5 |
| GS | Jersey Greensand | Fertrell, Bainbridge, PA | *MnO_2_, SiO_2_* | 0.10 |
| SB | Silica Blast® | Botanicare, Chandler, AZ | *Na_2_SiO_3_, K_2_SiO_3_* | none |
| IG | Ionic Grow® | Hydrodynamics International, Lansing, MI | *Ca(NO_3_)_2_, KNO_3_, H_3_PO_4_, HNO_3_, K_2_SO_4_* | 0.14 |
| EJ | Earth Juice® | Hydro-Organics, Chico, CA | *Kelp meal, MgSO_4_ borax, CoSO_4_, FeSO_4_, MnSO_4_, Na_2_MoO_4_, ZnSO_4_* | 1.45 |
| FF | Europonic® Fossil Fuel® | Hydrodynamics International, Lansing, MI | *Humic acids (Leonardite)* | 1.89 |
