# Supplementary material for: Plant growth conditions alter phytolith carbon
Source: Front Plant Sci. 2015 Sep 17;6:753. doi: 10.3389/fpls.2015.00753 (PMC4585121; doi:10.3389/fpls.2015.00753)
Supplement: Table S2 — Planter treatments by commercial amendments (adapted from Harutyunyan et al., 2014). [file Table2.DOC]

Table S2: Planter treatments by commercial amendments (adapted from Harutyunyan et al. 2014).

| **Planters** | | | | | | |
| --- | --- | --- | --- | --- | --- | --- |
|  | **A** | **B** | **C** | **D** | **E** | **F** |
| **Substrate** | Miracle Gro® | Greensand | Baked Sand | Baked Sand | Baked Sand | Baked Sand |
| **Nutrients** | In Miracle Gro® | Ionic® Grow | Ionic® Grow | Earth Juice®, IF^1^ | Fossil Fuel®, IF^1^ | IF^1^ |
| **Silica Provider** | In Miracle Gro® | In Greensand | Silica Blast | Silica Blast | Silica Blast | Silica Blast |

^1^ Inorganic fertilizer prepared "in-house".
